# The neutrophil‐to‐lymphocyte ratio as a marker of immunosenescence and COVID‐19 outcomes in the elderly: A narrative review

**DOI:** 10.14814/phy2.70682

**Published:** 2026-02-11

**Authors:** Maha Gasmi, Kimia Torabinasab, Ruth Williams‐Hooker, Santo Marsigliante, Antonella Muscella

**Affiliations:** ^1^ Higher Institute of Sport and Physical Education of Ksar Said Manouba University Tunis Tunisia; ^2^ Student Research Committee, Faculty of Nutrition and Food Technology Shahid Beheshti University of Medical Sciences Tehran Iran; ^3^ College of Health Sciences University of Memphis Memphis Tennessee USA; ^4^ Department of Biological and Environmental Science and Technologies (DiSTeBA) University of Salento Lecce Italy

**Keywords:** COVID‐19, immunosenescence, lymphopenia, neutrophil dysfunction, neutrophilia, neutrophil‐to‐lymphocyte ratio (NLR)

## Abstract

Older adults are highly vulnerable to severe COVID‐19. Unlike our previous work on broad immunosenescence, this review focuses on peripheral hematological markers as practical indicators of risk. To examine lymphopenia, neutrophilia, and the neutrophil‐to‐lymphocyte ratio (NLR) as clinically accessible markers of immune aging and COVID‐19 severity in older adults. Literature search of PubMed, Scopus, and Web of Science (up to 2025) for studies on aging, immunosenescence, lymphopenia, neutrophilia, NLR, and COVID‐19. These markers consistently correlate with worse COVID‐19 outcomes; NLR is a simple, reliable indicator of immune dysregulation, systemic inflammation, and mortality risk. Lymphopenia, neutrophilia, and elevated NLR are low‐cost, readily measurable markers associated with COVID‐19 severity, highlighting their prognostic value and complementing prior immunosenescence research.

## 1 INTRODUCTION

COVID‐19, caused by SARS‐CoV‐2, was declared a pandemic in March 2020 and led to over 15 million deaths globally, disproportionately affecting older adults with pre‐existing conditions (Blagosklonny, 2020; Chakraborty et al., 2021; Chen, Wu, et al., 2020; Chen, Zhou, et al., 2020; Gold, 2020; Ioannidis et al., 2021; Msemburi et al., 2023; Wu & McGoogan, 2020). Symptoms in the elderly ranged from mild to severe, with respiratory issues most frequent (Singhal et al., 2021; Wei et al., 2022). Neurological and cardiac symptoms were also common (Marghalani et al., 2023; Sousa Rêgo et al., 2023; Yassin et al., 2021). Aging‐related immunosenescence and chronic inflammation worsened COVID‐19 outcomes (Gasmi et al., 2025; Tizazu et al., 2025).

Aging profoundly affects the immune system through a process known as immunosenescence, which involves both adaptive and innate compartments. Thymic involution reduces naïve T‐cell output and diminishes adaptive immune responsiveness, resulting in lymphopenia, particularly decreased circulating T and B cells (Liang et al., 2024; Rubio‐Rivas et al., 2016). Simultaneously, chronic, low‐grade inflammation—or inflammaging—stimulates myelopoiesis and neutrophilia, with neutrophils exhibiting impaired chemotaxis and phagocytosis (Abdullah et al., 2025). The neutrophil‐to‐lymphocyte ratio (NLR) integrates these complementary changes, reflecting the shift toward innate immune dominance and serving as a widely available biomarker of systemic inflammation and immune imbalance (Kumarasamy et al., 2019). Unlike transient inflammatory markers or cytokine assays, lymphopenia, neutrophilia, and elevated NLR capture fundamental, age‐related immune remodeling and have consistently predicted COVID‐19 severity, hospitalization, and mortality in older adults (Awoke et al., 2023; Szklanna et al., 2021).

This narrative review aims to synthesize current evidence on lymphopenia, neutrophilia, and NLR in elderly COVID‐19 patients, linking these hematological changes to immunosenescence.

These hematological markers reflect key immunological shifts, such as the transition from adaptive to innate immunity, characteristic of immunosenescence (Kumarasamy et al., 2019). They are easily obtained from routine blood counts, making them accessible, cost‐effective, and clinically feasible (Liang et al., 2024). These markers are strongly linked to COVID‐19 severity and mortality in older adults, providing a robust framework to study aging, immune dysregulation, and disease prognosis (Rubio‐Rivas et al., 2016).

Unlike our and other previous works, which discussed aging‐associated immunological mechanisms and cytokine‐related alterations (Gasmi et al., 2025), the present review focuses on peripheral, quantitative hematological markers that are routinely available in clinical settings. By emphasizing their prognostic and translational value, this paper provides a complementary perspective that bridges laboratory findings on immunosenescence with practical tools for risk stratification and outcome prediction in older adults affected by COVID‐19.

## 2 LITERATURE SEARCH STRATEGY AND STUDY SELECTION

This narrative review was developed through a literature search in PubMed/MEDLINE, Scopus, and Web of Science. The literature search included studies from the earliest records to 2025, using the keywords and MeSH terms: aging, elderly, immunosenescence, lymphopenia, neutrophilia, neutrophil‐to‐lymphocyte ratio, hematological biomarkers, and COVID‐19.

**FIGURE 1:**
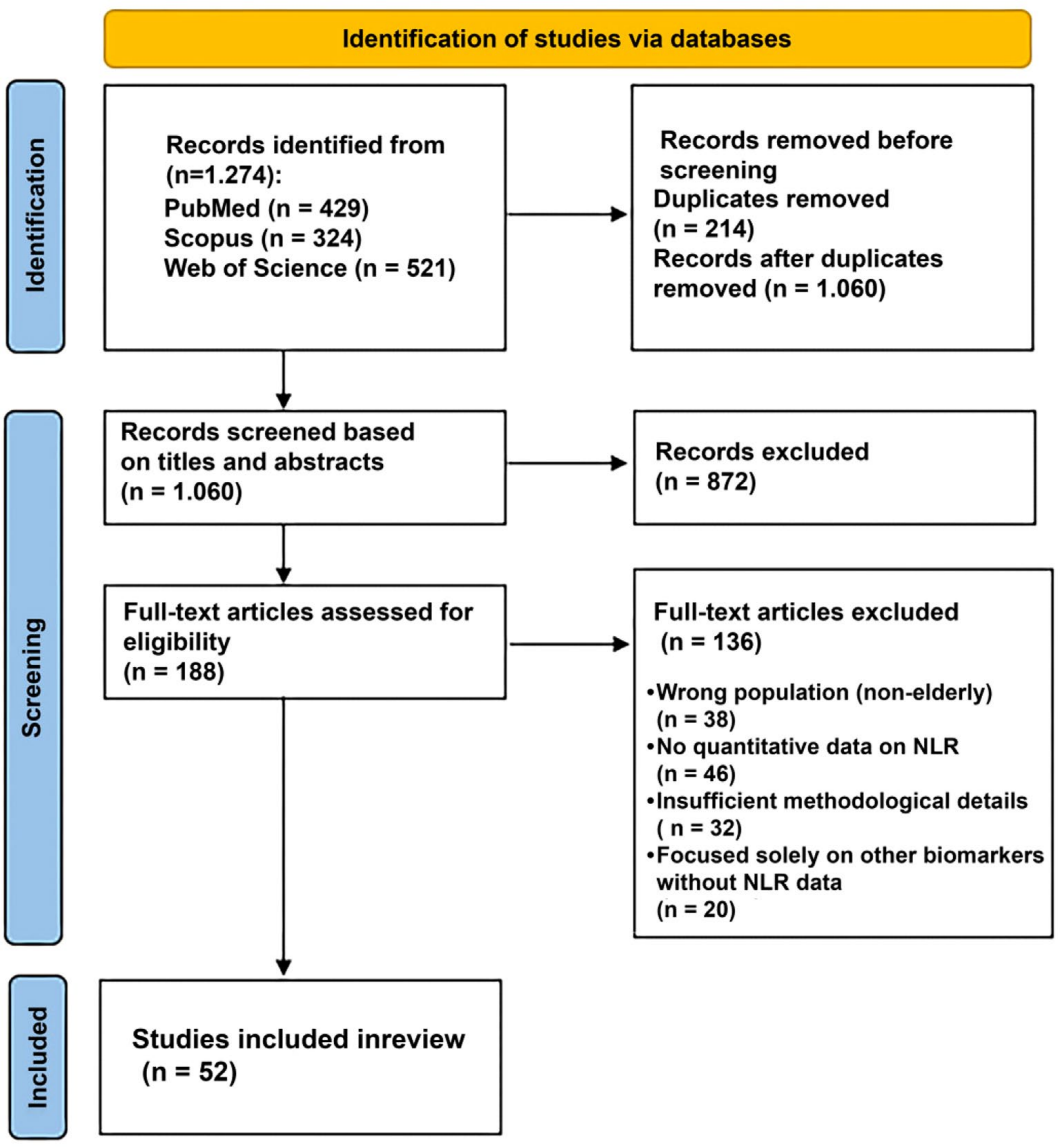
PRISMA 2020 flow diagram for the review, including the identification process, screening, and final number of studies included.

The study selection process is illustrated in Figure 1. Detailed methodology is in Appendix S1.

## 3 HEMATOLOGICAL PARAMETERS IN ELDERLY COVID‐19 PATIENTS: IMPLICATIONS AND PROGNOSTIC VALUE

Elderly patients with COVID‐19 often exhibit distinct hematological changes that are associated with worse outcomes (Charostad et al., 2023; Manaças et al., 2024). Common abnormalities include lymphopenia, neutrophilia, and an elevated neutrophil‐to‐lymphocyte ratio (NLR) (Crétel et al., 2011; Henry et al., 2020; Önal et al., 2022). These changes may reflect disease severity and help predict prognosis (Asaduzzaman et al., 2022; Nie et al., 2022; Zhou et al., 2020).

Comparative data on lymphocyte, neutrophil, and NLR thresholds in older adults are provided in Appendix S1, highlighting their prognostic relevance in COVID‐19 outcomes.

A summary of these findings is presented in Tables 1, 2, 3.

**TABLE 1:** Comparative of lymphopenia studies.

Author, year	Type of study	Participants characteristics	Methods	Main findings	Statistical values
Crétel et al. (2011)	Prospective observational study	Number: 51 elderly hospitalized patients Age: ≥75 years	Flow cytometry	41% had lymphopenia; 95% had abnormal phenotypes including inverted CD4/CD8 ratio	NR
Yan et al. (2010)	Observational study	Number: 80 healthy participants Age: 20‐ >80 years	Flow cytometry analysis	↓ in naïve CD8^+^ T cells, ↑ in memory/regulatory subsets, more pronounced in men	CD8^+^αβ^+^ T cells: p=0.0005 (males), p<0.0001 Effector memory T cells: p=0.0156 (sex difference) CD4:CD8 ratio: p=0.025 (sex difference)
Zidar et al. (2019)	Retrospective cohort study of	Number: 31178 patients Age: 30–63 years	Flow cytometry analysis	Lymphopenia thresholds: <1500/μL (relative), ≤1000/μL (severe). Lymphocyte ≤1500/μL associated with higher all‐cause mortality	Mild lymphopenia: HR = 1.3 (95% CI: 1.2–1.4) Severe lymphopenia: HR = 1.8 (95% CI: 1.6–2.1) High IH risk: HR = 3.2 (95% CI: 2.6–4.0) *p*‐value: <0.001 for overall association
Diao et al. (2020)	Retrospective observational study	Number: 522 COVID‐19 patients/healthy participants Age: 2–62 years Groups Non‐ICU care group ICU care group	Flow cytometry analaysis	Significant reductions in CD4^+^ and CD8^+^ T cells in severe cases	NR
Wang et al. (2020)	Experimental study	NK cells isolated from healthy adult donors Age: NR	Phenotypic analysis of T cell subsets.	↑ Glycolysis and ↑ OXPHOS; glycolysis essential for cytotoxicity (killing, degranulation, FasL); both pathways needed for IFN‐γ production	*p* < 0.05–0.001 for increased ECAR/OCR and IFN‐γ
Zhou et al. (2020)	Retrospective, multicentre cohort study	Number: 191 patients with COVID‐19 Age: 46–67 years Groups Non‐survivor group Survivor group	Complete blood count, real‐time RT‐PCR methods	Lymphopenia was strongly associated with increased mortality (*p* < 0.0001) and was a marker of disease severity	Older age: OR = 1.10 (95% CI 1.03–1.17), *p* = 0.0043 SOFA score: OR = 5.65 (95% CI 2.61–12.23), *p* < 0.0001 D‐dimer >1 μg/mL: OR = 18.42 (95% CI 2.64–128.55), *p* = 0.0033
Nie et al. (2022)	Retrospective study	Number: 136 ARDS patients Age: 61.8–65.1 years Groups Non‐survivor group Survivor group	Complete blood count which is performed by an automated hematology analyzer	Lymphopenia predicted ICU admission, mechanical ventilation, mortality	N/LPR: *p* = 0.003; OR: 8.934 ROC: AUC = 0.785, cut‐off = 10.57; KM: *p* < 0.001; NLR: *p* = 0.022; OR: 5.218; ROC: AUC = 0.679; Platelet count: *p* = 0.048; OR: 3.895; ROC: AUC = 0.326

Abbreviations: ↑, increase; ↓, decrease; ARDS, acute respiratory distress syndrome; AUC, area under curve; ECAR, extracellular acidification rate; IFN‐α, interferon‐alpha; N/LPR, neutrophil‐to‐lymphocyte and platelet ratio; NLR, neutrophil‐to‐lymphocyte ratio; NR, not reported; OCR, oxygen consumption rate; OXPHOS, oxidative phosphorylation; ROC, receiver operating characteristic; RT‐PCR, reverse transcription polymerase chain reaction; SOFA, sequential organ failure assessment.

**TABLE 2:** Prognostic value of neutrophilia in elderly COVID‐19 patients.

Author, year	Type of study	Participants characteristics	Methods	Main findings	Statistical values
Zhao et al. (2020)	Retrospective, observational study	Number: 539 patients with COVID‐19 Age:37–80 Groups Non‐survivor group Survivor group	RT‐PCR, complete blood count which is performed by an automated hematology analyzer	Non‐survivors had higher neutrophils (6.41 vs. 3.08 × 10^9^/L); admission neutrophilia independently predicted mortality (HR ≈ 4.4; 95% CI: 1.31–15.06; *p* = 0.017)	Univariable analysis (OR for death) Neutrophils: OR = 0.881 (0.790–0.982), *p* = 0.022 Lymphocytes: OR = 0.109 (0.032–0.186), *p* = 0.003 CD4^+^ T cells: OR = 1.005 (1.002–1.009), *p* = 0.002 Multivariable analysis (Generalized linear model) Neutrophils: *p* = 0.002 Lymphocytes: *p* = 0.016 CD4^+^ T cells: *p* = 0.048 ROC analysis (predictive accuracy and cut‐off) Neutrophils: AUC = 0.761, cut‐off = 5.835 × 10^9^/L Lymphocytes: AUC = 0.797, Cut‐off = 0.945 × 10^9^/L CD4^+^ T cells: AUC = 0.848, Cut‐off = 380.5 cells/μL
Henry et al. (2020)	Meta‐analysis study	Number: 4133 COVID‐19 patients Age: 25–87 years	Meta‐analysis	Admission neutrophilia associated with higher odds of severe disease (OR ≈ 7.99) and mortality (OR ≈ 7.87)	(AUC) and cut‐off Severe COVID‐19: AUC = 0.73 (95% CI: 0.58–0.88), cut‐off ≥14.5% (Sens: 81%, Spec: 64%) Severe AKI: AUC = 0.80 (95% CI: 0.68–0.92), cut‐off ≥14.6% (Sens: 65%, Spec: 92%) Need for RRT: AUC = 0.83 (95% CI: 0.68–0.97), cut‐off ≥14.6% (Adjusted OR) Severe COVID‐19: OR = 9.20 (95% CI: 1.04–81.74), *p* = 0.046 Severe AKI: OR = 16.03 (95% CI: 1.74–147.6), *p* = 0.014
Li, Chen, et al. (2020)	Systematic review and meta‐analysis	Number: 1579 patients Age: 39–69 years	Systematic review and meta‐analysis (78 studies)	Elevated WBC and neutrophils predicted mortality and ICU need; NLR correlated with severity	Disease severity Sensitivity (SEN)0.78 (95% CI: 0.70–0.84) Specificity (SPE)0.78 (95% CI: 0.73–0.83) Area under curve (AUC) 0.85 (95% CI: 0.81–0.88) Individual study cut‐offs: Varied from 3.0 to 13.4 Subgroup (cut‐off ≥4.5): AUC = 0.86 Subgroup (cut‐off <4.5): AUC = 0.82 Mortality Sensitivity (SEN)0.83 (95% CI: 0.75–0.89) Specificity (SPE)0.83 (95% CI: 0.74–0.89) Area under curve (AUC) 0.90 (95% CI: 0.87–0.92) Individual study cut‐offs: Varied from 3.0 to 11.8 Subgroup (cut‐off ≥6.5): AUC = 0.92 Subgroup (cut‐off <6.5): AUC = 0.84
Chen, Zhou, et al. (2020)	Retrospective, single‐center observational study	Number: 99 (2019‐nCoV pneumonia) patients Age: 21–82 years; mean age 55.5 ± 13.1 years; 67 men, 32 women; 51% with chronic diseases	RT‐PCR, complete blood count which is performed by an automated hematology analyzer. Chest X‐ray Chest CT	Neutrophilia was common at admission (38% of patients), often linked to severe inflammation or bacterial co‐infection; changes during hospitalization were not reported	NR
Asaduzzaman et al. (2022)	Retrospective, multicenter cohort study	Number: 245 patients Age: 70 ± 8.3 Groups Survivor group Non‐survivor group	Automated clinical chemistry & immunoassay analyzers Complete blood count which is performed by an automated hematology analyzer	Neutrophilia was a strong predictor of mortality in elderly hospitalized patients	Age (per year increase): OR = 1.05 (95% CI: 1.01–1.10), *p* = 0.009 Thrombocytopenia: OR = 3.56 (95% CI: 1.22–10.33), *p* = 0.019 Admission SpO_2_: OR = 0.91 (95% CI: 0.88–0.95), *p* = 0.001
El Azhary et al. (2025)	Retrospective study	Number: 119 patients Age: 44–60 years Groups Mild–Moderate group Severe–Critical group	Complete blood count which is performed by an automated hematology analyzer. Immunoassays/Clinical chemistry analyzers	Neutrophil (PNN) levels predict COVID‐19 severity: ↑ in neutrophil count (neutrophilia) was strongly associated with respiratory deterioration and the need for intubation	Cut‐off value: PNN‐Day 5 >7.7 × 10^9^/L Predictive power (AUC): Mortality: AUC = 0.951 ARDS: AUC = 0.974 Intubation: AUC = 0.951 Odds ratio (OR): Mortality: OR = 35.58 (95% CI: 6.57–192.64) ARDS: OR = 47.20 (95% CI: 7.81–285.09)
Liang et al. (2024)	Retrospective cohort study.	Number: 699 children Age: below 14 years of age. Groups Severe group and General group	Hematology and cell analysis Nucleic acid testing Coagulation analyzer	Neutrophil count was significantly associated with severe disease	Neutrophils: OR = 1.086 (95% CI: 1.054–1.119), *p* < 0.001 D‐dimer: OR = 1.005 (95% CI: 1.003–1.007), *p* < 0.001 Fibrinogen degradation products (FDP): OR = 1.341 (95% CI: 1.034–1.738), *p* = 0.027 B cells: OR = 1.076 (95% CI: 1.046–1.107), *p* < 0.001 Lactate dehydrogenase (LDH): OR = 1.008 (95% CI: 1.005–1.011), *p* < 0.001 Model performance: AUC = 0.974 (95% CI: 0.963–0.985); Hosmer–Lemeshow *p* = 0.547 (good calibration).
Middleton et al. (2020)	Prospective cohort study.	Number: 50 patients Age: 48.2–64.5 years Groups: Non‐ICU and ICU COVID‐19 infection group	Immunofluorescence staining for NETs and Platelets MPO‐DNA ELISA	COVID‐19 patients had significantly higher plasma levels of myeloperoxidase (MPO)–DNA complexes, a marker of neutrophil extracellular traps (NETs), compared with healthy controls	Intubation (*p* < 0.0001); Death (*p* < 0.0005); severity correlation (*p* = 0.0360); PaO_2_/FiO_2_ inverse correlation (*p* = 0.0340)

Abbreviations: ↑, increase; ↓, decrease; ARDS, acute respiratory distress syndrome; DNA, deoxyribonucleic acid; FDP, fibrinogen degradation; ICU, intensive care unit; LDH, lactate dehydrogenase; MPO, myeloperoxidase; NETs, marker of neutrophil extracellular traps; NLR, neutrophil‐to‐lymphocyte ratio; NR, not reported; PNN, polynuclear neutrophils; WBC, white blood cell count.

**TABLE 3:** Key studies on neutrophil‐to‐lymphocyte ratio (NLR) as prognostic marker in elderly COVID‐19 patients and related conditions.

Author, year	Type of study	Participants characteristics	Methods	Main findings	*p*‐value/OR/power
Fois et al. (2022)	Retrospective observational cohort study	Number:119 patients Age:57–85 years Groups Non‐survivor group Survivor group	Complete blood count which is performed by an automated hematology analyzer	Non‐survivors had significantly higher AISI, dNLR, NLPR, NLR, SII, and SIRI values compared to survivors	Systemic Inflammation Index (SII): HR = 1.0001 (95% CI: 1.0000–1.0001), *p* = 0.029
Liu et al. (2020)	Prospective cohort study	Number: 115 patients Age: 1–92 years Groups Patients with COVID‐19 infection group Patients in the validation cohort	RT‐PCR Clinical chemistry & immunoassay analyzers, complete blood count which is performed by an automated hematology analyzer	Neutrophil‐to‐lymphocyte ratio (NLR) was identified as the strongest independent risk factor for predicting critical illness in COVID‐19 patients	Neutrophil‐to‐lymphocyte ratio (NLR): identified as an independent risk factor for critical illness. AUC (derivation cohort): 0.849 (95% CI: 0.707–0.991) AUC (validation cohort): 0.867 (95% CI: 0.747–0.944) Cut‐off value: NLR ≥3.13 in patients aged ≥50 predicted 50% incidence of critical illness (vs. 9.1% with NLR <3.13)
Li, Liu, et al. (2020)	Systematic review and meta‐analysis	Number: 1579 patients Age: 39–69 years	Systematic review and meta‐analysis (78 studies)	(NLR) has high predictive accuracy for both disease severity and mortality in COVID‐19 patients. Measuring NLR on admission can help clinicians identify high‐risk patients early, prioritize intensive care, and reduce overall mortality	Disease severity Sensitivity (SEN) 0.78 (95% CI: 0.70–0.84) Specificity (SPE) 0.78 (95% CI: 0.73–0.83) Area under curve (AUC) 0.85 (95% CI: 0.81–0) Mortality Sensitivity (SEN) 0.83 (95% CI: 0.75–0.89) Specificity (SPE) 0.83 (95% CI: 0.74–0.89) Area under curve (AUC) 0.90 (95% CI: 0.87–0.92)
Di Rosa et al. (2023)	Multicenter prospective cohort study	Number:1214 hospitalized geriatric patients Age: >65 years	Neutrophil‐to‐lymphocyte ratio (NLR) measured from routine blood tests on admission	Higher NLR at admission predicted mortality independent of the admission diagnosis	HR = 1.06 (95% CI: 1.04–1.08), *p* < 0.001
Yıldız et al. (2023)	Single‐center cross‐sectional and observational study	Number: 152 hospitalized moderate‐to‐severe COVID‐19 patients Age: Mean 58.2 ± 13.7 years (64 female, 88 male)	Patients were classified as low, moderate, or high risk based on D‐dimer and NLR cut‐off values.	High‐risk PRI‐COVID (D‐dimer >1.07 μg/mL and NLR >3.83) was associated with a 6.37‐fold higher 30‐day mortality risk and a 5.82‐fold higher overall mortality risk compared with the low/moderate group. Both D‐dimer and NLR were independent predictors of mortality.	Mortality D‐dimer Sensitivity (SEN): 0.68 Specificity (SPE): 0.80 AUC: 0.75 (±0.05), *p* < 0.001 NLR Sensitivity (SEN): 0.92 Specificity (SPE): 0.49 AUC: 0.73 (±0.05), *p* < 0.001 Combined PRI‐COVID model HR = 6.37 (*p* < 0.001) for 30‐day mortality HR = 5.82 (*p* < 0.001) for overall mortality
Alonso Batun et al. (2025)	Retrospective cohort study	Number: 172 hospitalized adult COVID‐19 patients (severe or critical)	Clinical, demographic, and laboratory variables analyzed; univariate and multivariate logistic regression; ROC analysis	NLR ≥9.76 identified as a strong independent predictor of in‐hospital mortality; high predictive accuracy for mortality	OR = 1.66 (95% CI: 1.26–2.17), *p* < 0.001 Mortality prediction: Sensitivity = 0.83 (95% CI: 0.75–0.89) Specificity = 0.83 (95% CI: 0.74–0.89) AUC = 0.85 (95% CI: 0.81–0.88)
Önal et al. (2022)	Retrospective observational cohort study.	Number: 100 patients with COVID‐19 Age: 72.13–73.14 years	RT‐qPCR Complete blood count which is performed by an automated hematology analyzer.	Elevated NLR associated with severity, ventilation need, and mortality	NLR: OR = 1.371 (95% CI: 1.067–1.761), *p* = 0.014; cut‐off >7.8 (83.3% sens., 97.7% spec.) LDH: OR = 1.011 (95% CI: 1.001–1.023), *p* = 0.047; cut‐off >300 U/L (100% sens., 79.3% spec.)
Sarengat et al. (2021)	Cross‐sectional study	Number: 21 patients with acute thrombotic stroke and COVID‐19 (12 males, 9 females) Age: 37–78 years	RT‐PCR Complete blood count which is performed by an automated hematology analyzer. Non‐contrast head computerized tomography scan (CT‐scan)	Higher NLR values were associated with greater stroke severity in patients with COVID‐19–related acute thrombotic stroke.	*r* = 0.45, *p* = 0.041
Nie et al. (2022)	Retrospective study	Number: 136 ARDS patients Age: 61.8–65.1 years Groups Non‐survivor group Survivor group	Complete blood count which is performed by an automated hematology analyzer.	The neutrophil‐to‐lymphocyte and platelet ratio (N/LPR) is a strong, independent biomarker for predicting 28‐day mortality in ARDS, outperforming traditional markers like NLR or platelet count alone.	N/LPR: *p* < 0.05 (multivariable); ROC: AUC = 0.785, cut‐off = 10.57 (sens 74.6%, spec 72.5%); KM: *p* < 0.001. NLR: *p* < 0.05; ROC: AUC = 0.679. Platelet count: *p* < 0.05; ROC: AUC = 0.326
Bota et al. (2024)	Retrospective cohort study	Number: 138 elderly patients Age: 52.5–83.7 years Groups Elderly patients group Control patients group	Complete blood count which is performed by an automated hematology analyzer. Clinical biochemistry analyzers	High SII and dNLR values at admission indicate a higher risk of severe COVID‐19, ICU admission, and mortality in patients aged ≥80 years.	SII: AUC = 0.857 (95% CI: 0.795–0.919), *p* < 0.001; cutoff = 920 × 10^9^/L (86% sens., 78% spec.); HR = 2.9 for ICU (95% CI: 1.8–4.6, *p* < 0.001); HR = 3.2 for mortality (95% CI: 1.9–5.2, *p* < 0.001) dNLR: AUC = 0.792 (95% CI: 0.722–0.862), *p* < 0.001
Arai et al. (2023)	Retrospective cohort study.	Number: 86 patients diagnosed with AE‐IPF Age: 66.0–75.25 years	Complete blood count which is performed by an automated hematology analyzer. Enzyme‐linked immunosorbent assay (ELISA)	NLR at admission (Day‐1) is an independent predictor of 90‐day mortality. NLR on Days 4 and 8 predicts survival in patients without oxygenation deterioration.	Day‐1 NLR >12.13 AUC = 0.712 (95% CI: 0.602–0.823) HR = 2.906 (95% CI: 1.635–5.166) *p* < 0.001 Day‐4 NLR > 14.90 AUC = 0.684 (95% CI: 0.567–0.801) *p* < 0.001 Day‐8 NLR > 10.56 AUC = 0.774 (95% CI: 0.666–0.883) HR = 3.927 (95% CI: 1.685–8.267) • *p* < 0.001
Ciccullo et al. (2020)	Letter to the Editor	Number: 74 COVID‐19 patients Age: 52–73 years.	Meta‐analysis	Confirmed neutrophilia strongly associated with worse outcomes, including severity and death	Age (severe vs. non‐severe) *p* = 0.007. NLR (severe vs. non‐severe) *p* = 0.001. Younger associated with clinical improvement: *p* = 0.040; NLR <3 associated with clinical improvement: *p* = 0.010. NLR >4 predicts ICU admission: *p* = 0.046. Older age predicts death: *p* = 0.047
Ince et al. (2024)	Cohort study	Number: 204 COVID‐19 patients Age: 66–76 years Groups Survivors who recovered from their intensive care unit (ICU) stay (Group 1) Patients who died (Group 2)	RT‐PCR, complete blood count which is performed by an automated hematology analyzer. Clinical biochemistry assays	Elevated inflammatory markers (procalcitonin, NLR, PLR, FL index) are associated with higher mortality risk in ICU COVID‐19 patients and can be used to predict outcomes and optimize patient care.	Age >73 → OR = 2.1, *p* = 0.05 Procalcitonin >0.35 ng/mL → OR = 5.6, *p* < 0.001 FL index >1228 mg/dL → OR = 3.5, *p* = 0.003 PLR >212 → OR = 3.5, *p* = 0.030 NLR >5.8 → OR = 1.6, *p* = 0.043

Abbreviations: ↑, increase; ↓, decrease; AISI, aggregate index of systemic inflammation; ARDS, acute respiratory distress syndrome; CBC, blood cell count; dNLR, derived neutrophil to lymphocyte ratio; FL index, ferritin lactate index; MLR, monocyte to lymphocyte ratio; MPR, mean platelet volume to platelet ratio; N/LPR, neutrophil to lymphocyte × platelet ratio; N/LPR, neutrophil‐to‐lymphocyte and platelet ratio; NLR, neutrophil to lymphocyte ratio; NR, not reported; PLR, platelet to lymphocyte ratio; PLR, platelet‐to‐lymphocyte ratio; RT‐qPCR, reverse transcription quantitative polymerase chain reaction; SII, systemic inflammation index; SIRI, systemic inflammation response index.

### 3.1 Lymphopenia

Lymphocytes, especially T and B cells, are central to adaptive immunity (Alberts et al., 2002). Aging causes thymic involution, reducing naïve T cells and increasing memory and senescent cells (Fujimori & Ohigashi, 2024; Palmer et al., 2018). Functional decline of CD8^+^ T cells, along with elevated IL‐6 and TNF‐α, impairs immune response and triggers lymphocyte apoptosis (Zhou et al., 2022; Velazquez‐Salinas et al., 2019); while CD4^+^ remains stable (Lin et al., 2016; Valiathan et al., 2016). Oxidative stress and telomere shortening limit naïve T cell expansion (Anderson et al., 2022), while shifts in hematopoiesis deplete CD4^+^/CD8^+^ cells (Henry et al., 2020; Maeda et al., 2009), disrupting immune balance in the elderly (Crétel et al., 2011).

Early observations showed lymphocyte counts falling from ~5000/μL in adulthood to ~1500/μL by age 90, with stable granulocytes (MacKinney, 1978). Lymphopenia predicts frailty, longer hospitalization, and higher mortality (Rubio‐Rivas et al., 2016; Zafrir et al., 2022; Zidar et al., 2019). Thymic involution reduces naïve T‐cell output (Fujimori & Ohigashi, 2024; Palmer et al., 2018), while functional deterioration leads to loss of polyfunctional T cells (Van Epps et al., 2014). In geriatric wards, 41% of patients aged ≥75 years presented with lymphopenia, and 95% exhibited abnormal lymphocyte phenotypes, including inverted CD4/CD8 ratios, consistent with findings reported in patients with multimorbidity (Crétel et al., 2011). Lifespan studies confirmed reductions in CD3^+^, CD4^+^, CD8^+^, and CD19^+^ cells with increased IL‐6/TNF‐α (Valiathan et al., 2016). Flow cytometry revealed declines in naïve/CD8^+^ T cells and increases in memory/regulatory subsets, especially in men (Yan et al., 2010). B cell development is also impaired due to intrinsic aging and reduced hematopoietic stem cell potential (Cancro, 2020; Cancro et al., 2009; Frasca et al., 2020; Guerrettaz et al., 2008; Stephan et al., 1997).

Clinically, lymphopenia (<1.0–1.1 × 10^9^/L) is linked to reduced independence, cognitive impairment (Dennis et al., 1998), longer hospital stays, and higher mortality (Rubio‐Rivas et al., 2016). Population studies (>31,000 adults) confirmed increased all‐cause mortality with lymphocyte ≤1500/μL (Zidar et al., 2019); similar findings emerged in coronary angiography cohorts (Zafrir et al., 2022).

**FIGURE 2:**
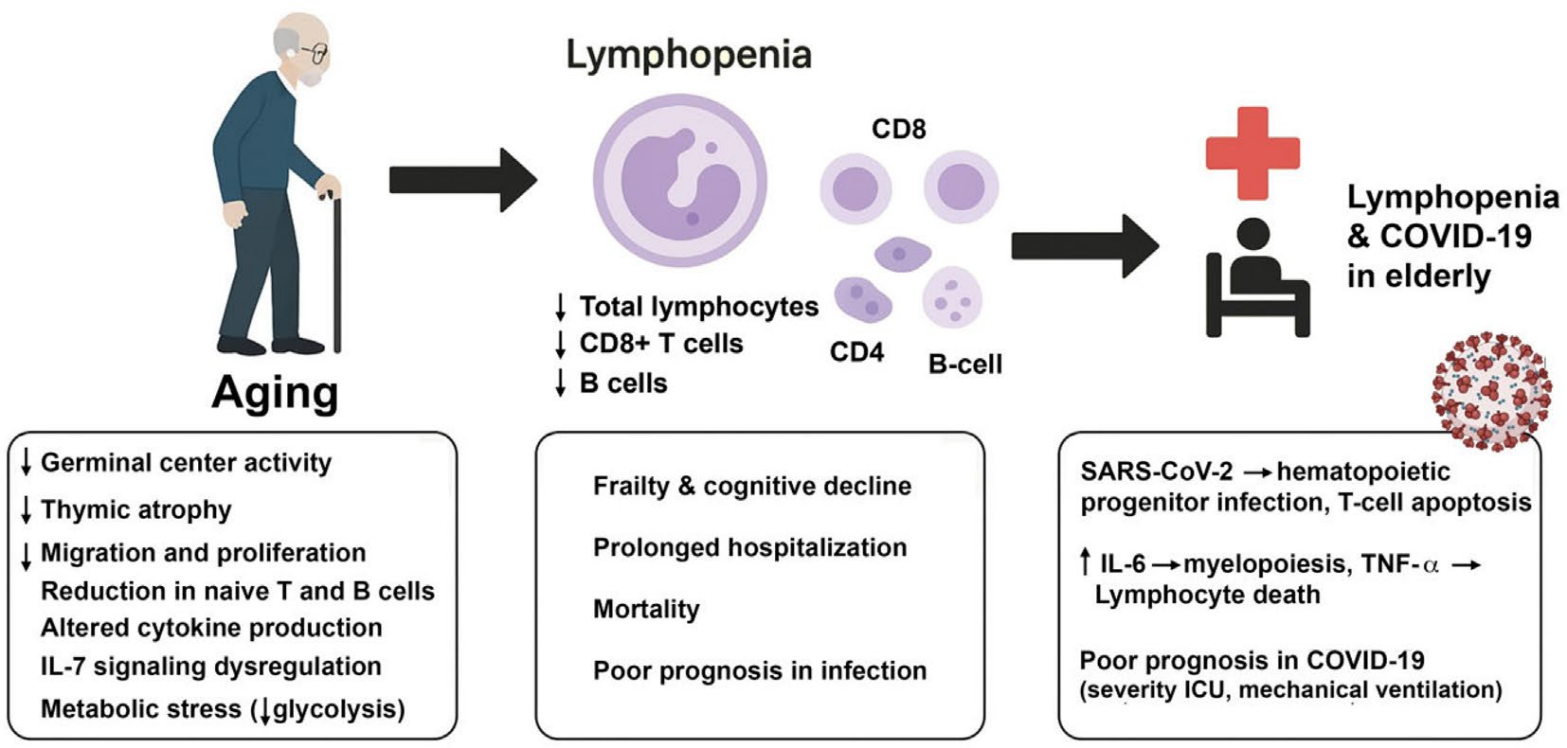
Lymphopenia in elderly patients with COVID‐19. Aging and SARS‐CoV‐2 infection contribute to reduced lymphocyte counts and impaired function. Mechanisms include thymic atrophy, telomere attrition, altered cytokine signaling (IL‐6, TNF‐α), progenitor cell infection, and T‐cell apoptosis. Consequences are reduced immune competence, frailty, prolonged hospitalization, poor prognosis, and increased mortality.

COVID‐19 further highlighted lymphopenia. It is frequent in elderly patients (Guan et al., 2020; Huang & Pranata, 2020; Tavakolpour et al., 2020), and pre‐existing lymphopenia increases severe disease risk (Garbo et al., 2022) (Figure 2). Reports show prevalence of 40%–63% in hospitalized adults (Huang et al., 2020; Zhou et al., 2020). A meta‐analysis of 21 studies (*n* = 4133) confirmed higher odds of severe disease and mortality (Henry et al., 2020). Observational studies linked lymphopenia with greater severity, ICU admission, mechanical ventilation, dialysis, and mortality (Garyali et al., 2025; Liu et al., 2020; Niu et al., 2022; Yuan et al., 2021). Functional impairments include reduced CD4^+^/CD8^+^ T cells (Diao et al., 2020), fewer naïve but more senescent T cells (Wang et al., 2020) and altered CD4/CD8 ratios and Tregs (Wang et al., 2021).

Sex differences show men have lower lymphocyte percentages and higher IL‐6/IL‐10 levels (Ghaab et al., 2024). Male aging accelerates the decline in naïve/CD4^+^ T cells (Calabrò et al., 2023). Mechanistically, SARS‐CoV‐2 may infect hematopoietic stem/progenitor cells via ACE2/CD209L, impair lymphoid differentiation (Ropa et al., 2021; Shouman et al., 2024), trigger inflammasome‐mediated pyroptosis (Kucia et al., 2021; Zhao et al., 2020), and skew hematopoiesis toward myelopoiesis (De Bruin et al., 2014). CD71^+^ erythroid cells expressing viral entry proteins also suppress adaptive responses (Shahbaz et al., 2021). Splenic/lymph node infection adds to tissue damage (Li, Liu, et al., 2020; Shaukat et al., 2021). CD147 may mediate T‐cell entry (Kuklina, 2022; Ragotte et al., 2021; Wang et al., 2020; Wu et al., 2022). Telomere attrition also limits the expansion of naïve T cells (Anderson et al., 2022). scRNA‐seq revealed T/NK exhaustion, myeloid activation, and cytokine decline (Lewis et al., 2021). Age drives reductions in naïve T cells independently of COVID‐19 (Sturmlechner et al., 2025), and male sex correlates with lower CD3^+^/CD4^+^ counts (Löhr et al., 2023). Severe infection leads to immune exhaustion, cytokine storm, ARDS, and multiorgan failure (Gu et al., 2005; Melo et al., 2021; Chen, Wu, et al., 2020; Chen, Zhou, et al., 2020). IL‐6 shifts hematopoiesis toward myelopoiesis (Maeda et al., 2009; Velazquez‐Salinas et al., 2019); TNF‐α drives lymphocyte death (Álvarez et al., 2011; Hampton & Chtanova, 2019; Henry et al., 2020; Li & Beg, 2000). IFN‐γ promotes PD‐L1 expression and suppresses proliferation (Cautivo et al., 2022; De Kleijn et al., 2013; Peñaloza et al., 2021; Vafadar Moradi et al., 2021). Cytokines and chemokines also drive exhaustion (Yang et al., 2022).

Metabolic stress drives immune cells to shift toward aerobic glycolysis, which increases lactate production and extracellular acidity (Doughty et al., 2006; Krawczyk et al., 2010; Pearce et al., 2013; Rodríguez‐Espinosa et al., 2015; Soto‐Heredero et al., 2020; Wang et al., 2020). In parallel, glutaminolysis supports cellular energy demands (Macintyre et al., 2014), whereas glutamine deficiency impairs effector T‐cell function and promotes regulatory T cell (Treg) expansion (Nakaya et al., 2014). However, Tregs may fail to fully suppress inflammation under certain conditions (De Waal Malefyt et al., 1991). In the context of viral infection, SARS‐CoV‐2 ORF3a has been shown to upregulate HIF‐1, thereby amplifying pro‐inflammatory responses (Farshbafnadi et al., 2021; Tian et al., 2021). Moreover, hypoxia further compromises T‐cell proliferation by disrupting STAT5a signaling (Gaber et al., 2013). Apoptosis also underlies lymphopenia: coronaviruses induce caspase‐dependent T‐cell death (Boonnak et al., 2014; Chu et al., 2016; Law et al., 2005; Yang et al., 2005). Elevated Fas/PD‐1 associates with exhaustion (André et al., 2022). Biomarker and transcriptomic data confirm apoptosis pathways (André et al., 2022; Xiong et al., 2020; Zhu et al., 2020). Mitochondrial VDAC1^+^ subsets undergo apoptosis (Thompson et al., 2021). Oxidative stress genes also promote apoptosis (Shen et al., 2022). In vitro, T‐cell apoptosis can be triggered by monocyte‐derived cytokines (Pontelli et al., 2022).

Thus, lymphopenia is a key feature of aging and COVID‐19, linked to frailty, severe disease, and poor outcomes. Age‐ and sex‐related lymphocyte decline, functional impairments, and virus‐induced mechanisms converge to reduce immune competence in older adults.

### 3.2 Neutrophil dysfunction in aging and neutrophilia

Neutrophils are key phagocytes in infection control and tissue repair, recruited by cytokines/chemokines. Their numbers remain stable with age, but elderly individuals show impaired chemotaxis, phagocytosis, and reduced NET‐mediated killing (Aroca‐Crevillén et al., 2024; Butcher et al., 2001; Hazeldine et al., 2014; Qian et al., 2014; Sabbatini et al., 2022; Wenisch et al., 2000). Dysfunction relates to decreased CD16 expression (Butcher et al., 2001), reduced migration to GM‐CSF/fMLP (Hajishengallis, 2010), altered PI3K signaling (Sapey et al., 2014), and lower ROS/glucose uptake (Wenisch et al., 2000). Chronic inflammation shows delayed apoptosis and impaired turnover (McCracken & Allen, 2014). Aged neutrophils have reduced ATP (Richer et al., 2018) and impaired purinergic signaling (Whyte et al., 1993).

Age also weakens TLR2/MyD88 and TREM‐1 signaling (Hajishengallis, 2010). Neutrophil ROS induce telomere damage, promoting inflammaging (Jacome Burbano et al., 2021). Although not directly causal, dysfunction contributes to age‐related disease progression (Van Avondt et al., 2023). Neutrophilia (>7.5 × 10^9^/L) is a prognostic biomarker in older adults. Meta‐analyses show admission neutrophilia associated with increased severity (OR ≈ 7.99) and mortality (OR ≈ 7.87) (Ciccullo et al., 2020; Henry et al., 2020). In elderly COVID‐19 patients, neutrophilia may indicate bacterial co‐infection. In a cohort (mean age ≈70), non‐survivors had higher neutrophils (6.41 vs. 3.08 × 10^9^/L) (Zhao et al., 2020); admission neutrophilia independently predicted mortality (HR ≈ 4.4; 95% CI: 1.31–15.06; *p* = 0.017) (Zhao et al., 2020).

**FIGURE 3:**
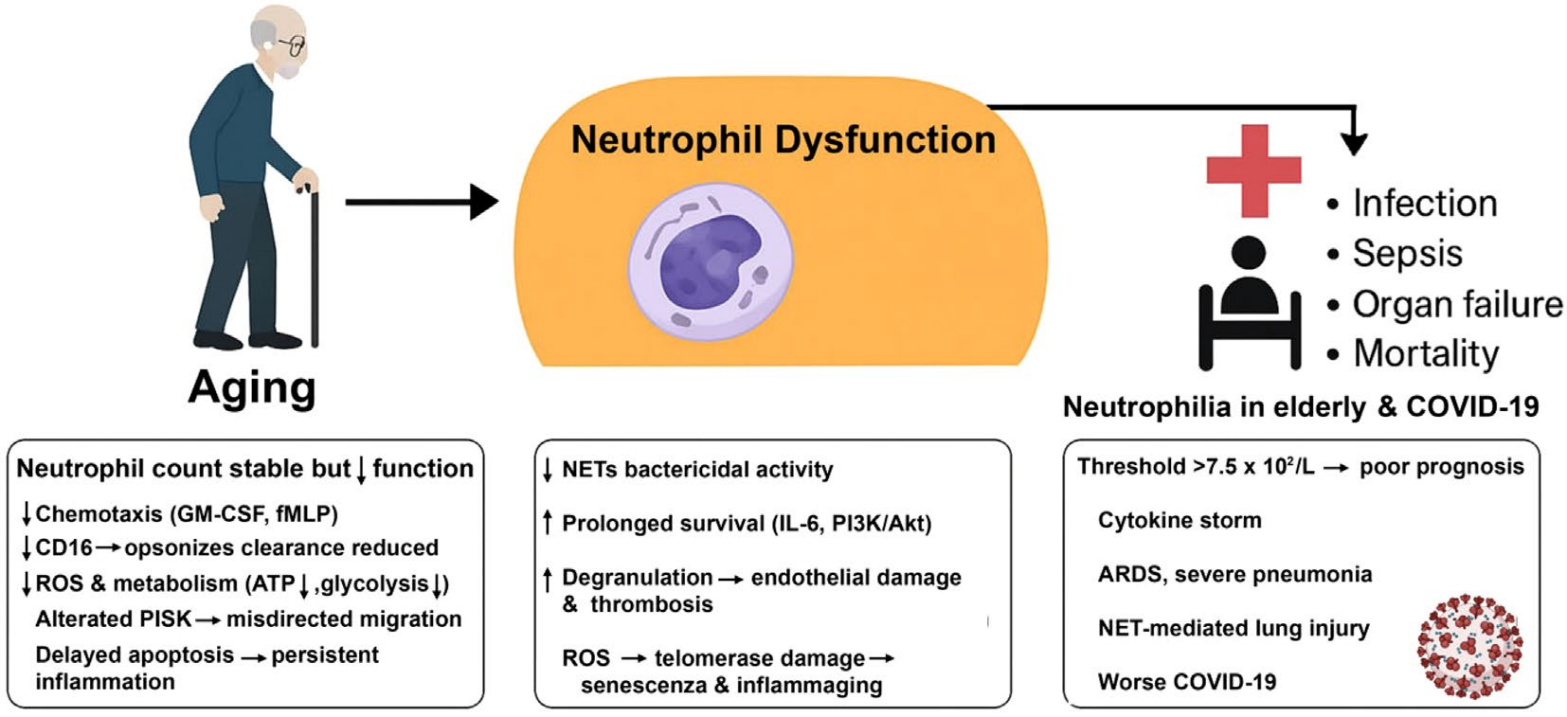
Neutrophil dysfunction in aging and its clinical consequences in elderly patients with COVID‐19. Aging is associated with impaired chemotaxis, phagocytosis, NET and ROS production, reduced ATP levels, altered PI3K/AKT signaling, and delayed apoptosis. These alterations, together with neutrophilia, contribute to a pro‐inflammatory environment characterized by chronic low‐grade inflammation (inflammaging), increased susceptibility to infections, tissue damage mediated by ROS, cytokine storm (COVID‐19), ARDS, and higher mortality.

Neutrophilia reflects hyperinflammation and cytokine storm (Cavalcante‐Silva et al., 2021). In elderly COVID‐19, neutrophils correlate with severity (Ince et al., 2024). IL‐6 prolongs survival via JAK–STAT and PI3K‐AKT signaling; aged neutrophils show PI3K hyperactivation, misdirected migration, and inflammation (Larbi et al., 2005). In COVID‐19, they display excessive degranulation, cytokine storms, endothelial damage, thrombosis (Gullotta et al., 2023) and promote ARDS via tissue injury and vascular permeability. These alterations, together with neutrophilia, sustain chronic low‐grade inflammation (inflammaging) and worsen COVID‐19 severity, as shown in Figure 3.

A meta‐analysis of 78 studies confirmed admission leukocytosis and neutrophilia predict mortality, ICU need, and correlate with NLR (Li, Liu, et al., 2020). In ≥60‐year‐old hospitalized patients, neutrophilia remained an independent predictor of death (HR ≈ 4.4) along with dyspnea, age, and troponin (Zhao et al., 2020). Meta‐analyses again confirmed associations with severity and mortality (Ciccullo et al., 2020; Henry et al., 2020).

Qualitative alterations also contribute to neutrophilia and leukocytosis that characterize cytokine storm, with pulmonary infiltration and NET‐mediated injury (Middleton et al., 2020). Further, neutrophilia predicted mortality in elderly COVID‐19 cohorts from Bangladesh (Asaduzzaman et al., 2022) and other populations (El Azhary et al., 2025; Liang et al., 2024). Neutrophil count at Day 5 predicted deterioration (AUC up to 0.974 for ARDS) (Chen, Zhou, et al., 2020). Overall, admission and early neutrophilia are robust biomarkers of poor outcomes, stressing the need for monitoring and early intervention.

### 3.3 Neutrophil‐to‐lymphocyte ratio in elderly COVID‐19 patients

**FIGURE 4:**
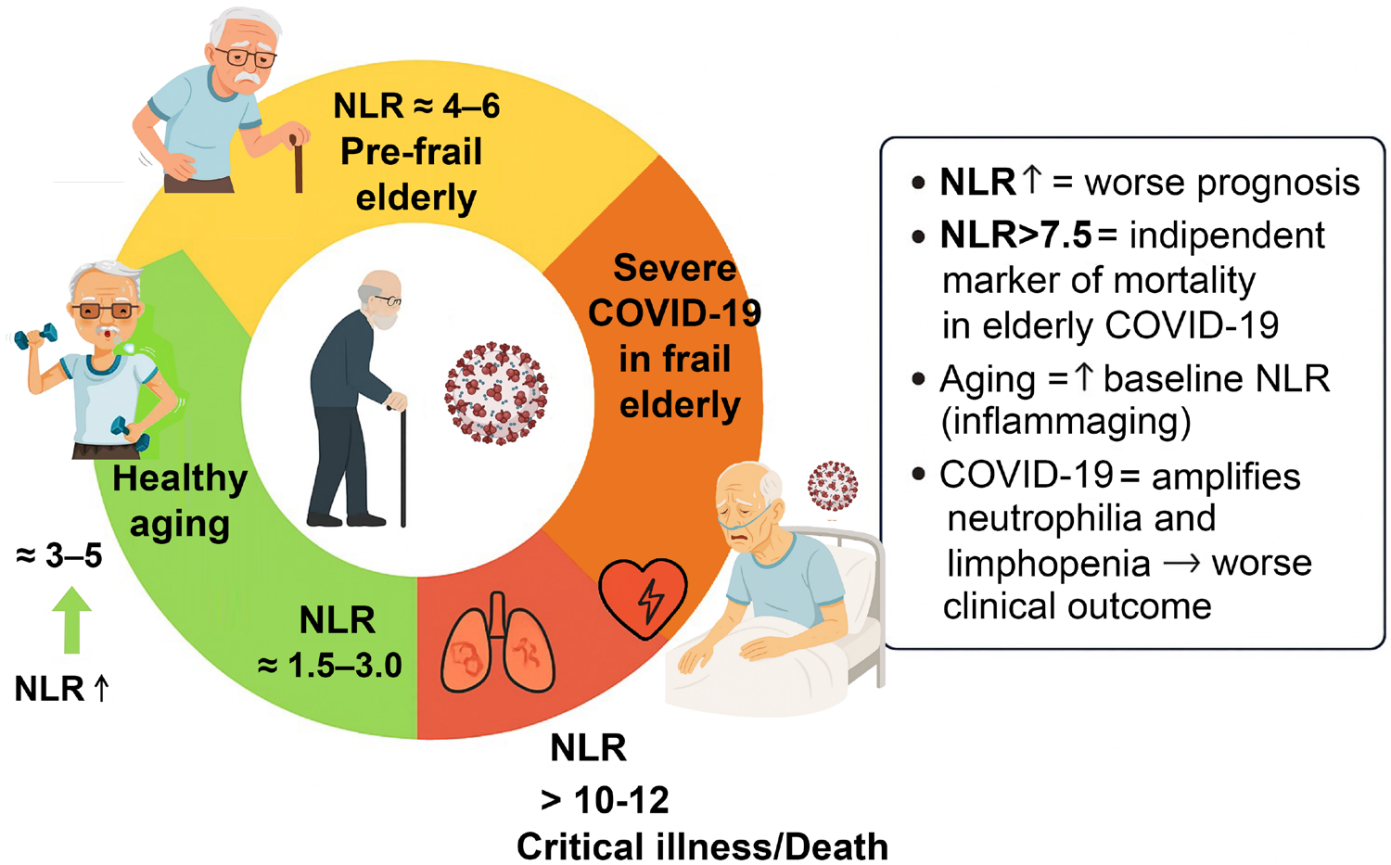
Prognostic role of the neutrophil‐to‐lymphocyte ratio (NLR) in elderly patients with COVID‐19. Elevated NLR is a marker of systemic inflammation and immunosenescence, and is associated with increased disease severity, need for intensive care, acute respiratory distress syndrome (ARDS), and higher mortality.

The neutrophil‐to‐lymphocyte ratio (NLR) has emerged as a robust and readily accessible prognostic marker in older patients with COVID‐19, reflecting the interplay between lymphopenia and neutrophilia during systemic inflammation (Önal et al., 2022). Elevated NLR values have been consistently associated with increased disease severity, the need for mechanical ventilation, and mortality, highlighting its utility as a simple, rapid, and cost‐effective tool for early risk stratification in clinical practice (El Azhary et al., 2025; Önal et al., 2022; Ulloque‐Badaracco et al., 2021) (Figure 4).

Several clinical cohorts have confirmed the prognostic significance of NLR in elderly patients. Fois et al. (2022) demonstrated in a multicenter Italian cohort that elevated NLR at admission independently predicted 30‐day mortality. Similarly, Liu et al. (2020) identified an NLR cut‐off ≥3.13 as a reliable predictor of severity and mortality in hospitalized patients with a mean age above 60 years, while Li, Liu, et al. (2020) confirmed in a multicenter study that both NLR and PLR were strong predictors of progression to severe disease in patients aged ≥60 years. More recently, Di Rosa et al. (2023) showed that incorporation of NLR into multivariate prognostic models significantly improved prediction of in‐hospital mortality in elderly Italian cohorts, and Yıldız et al. (2023) reported that elevated NLR at hospital admission was strongly associated with higher in‐hospital mortality and need for invasive ventilation in patients aged ≥65 years. Supporting these findings, Alonso Batun et al. (2025), in a cohort of 172 severe or critically ill hospitalized COVID‐19 patients, identified an NLR ≥9.76 as a strong independent predictor of in‐hospital mortality with high predictive accuracy. In hospitalized elderly populations, NLR measured at admission or during hospitalization has also demonstrated strong predictive value for adverse outcomes, including acute respiratory distress syndrome (ARDS), intensive care unit (ICU) admission, and high mortality (Sarengat et al., 2021; Yoon & Lee, 2021; Nie et al., 2022).

Multicenter cohorts of patients aged ≥60 years have confirmed that elevated NLR, along with derived indices such as dNLR and platelet‐to‐lymphocyte ratio (PLR), represent some of the strongest laboratory predictors of progression to critical illness and death, independent of frailty status (Bota et al., 2024; Li, Chen, et al., 2020; Lian et al., 2020). In very old patients (≥80 years), composite systemic inflammation indices including the systemic immune‐inflammation index (SII) and derived NLR have demonstrated strong predictive performance for mortality (Bota et al., 2024; Li, Liu, et al., 2020; Zhang et al., 2024). Large meta‐analyses encompassing over 15,000 COVID‐19 patients have further shown that each unit increase in NLR significantly elevates the odds of severe disease and all‐cause mortality, with predictive value maintained across studies with low risk of bias (Ulloque‐Badaracco et al., 2021).

Beyond acute infection, elevated baseline NLR has been shown to independently predict all‐cause and cardiovascular mortality in community‐dwelling older adults, and longitudinal increases correlate with multimorbidity accumulation and higher risk of death (Paganelli & Di Iorio, 2025; Pellegrino et al., 2024). In acute care settings, including elderly patients with community‐acquired pneumonia or acute medical conditions, NLR thresholds above 8 have been associated with markedly increased 30‐day mortality, and incorporation of NLR into severity scoring systems such as SMARTCOP improves prognostic accuracy (Huang et al., 2025). Similar predictive value has been reported in elderly septic patients with diabetes, where an NLR threshold of 3.5 independently predicted 90‐day mortality beyond conventional severity indices (Zhang et al., 2024).

Interestingly, the prognostic utility of NLR extends also to other acute conditions in the elderly. Arai et al. (2023) demonstrated that day‐1 NLR was a strong predictor of 90‐day survival in patients with acute exacerbation of idiopathic pulmonary fibrosis (AE‐IPF), suggesting a broader role of NLR as a marker of systemic inflammatory burden beyond COVID‐19. Finally, in COVID‐19‐related thrombotic stroke, NLR has shown a positive correlation with National Institutes of Health Stroke Scale (NIHSS) scores, reinforcing its clinical utility in predicting severe complications (Sarengat et al., 2021).

Collectively, these findings underscore the value of NLR and its derivatives as reliable, cost‐effective prognostic markers in aging populations, spanning community‐dwelling older adults, hospitalized patients with COVID‐19, sepsis, pneumonia, or multimorbidity, and even those with non‐infectious respiratory exacerbations such as AE‐IPF (Asaduzzaman et al., 2022; Hazeldine & Lord, 2021; Liang et al., 2024; Liu et al., 2020).

## 4 DISCUSSION

**FIGURE 5:**
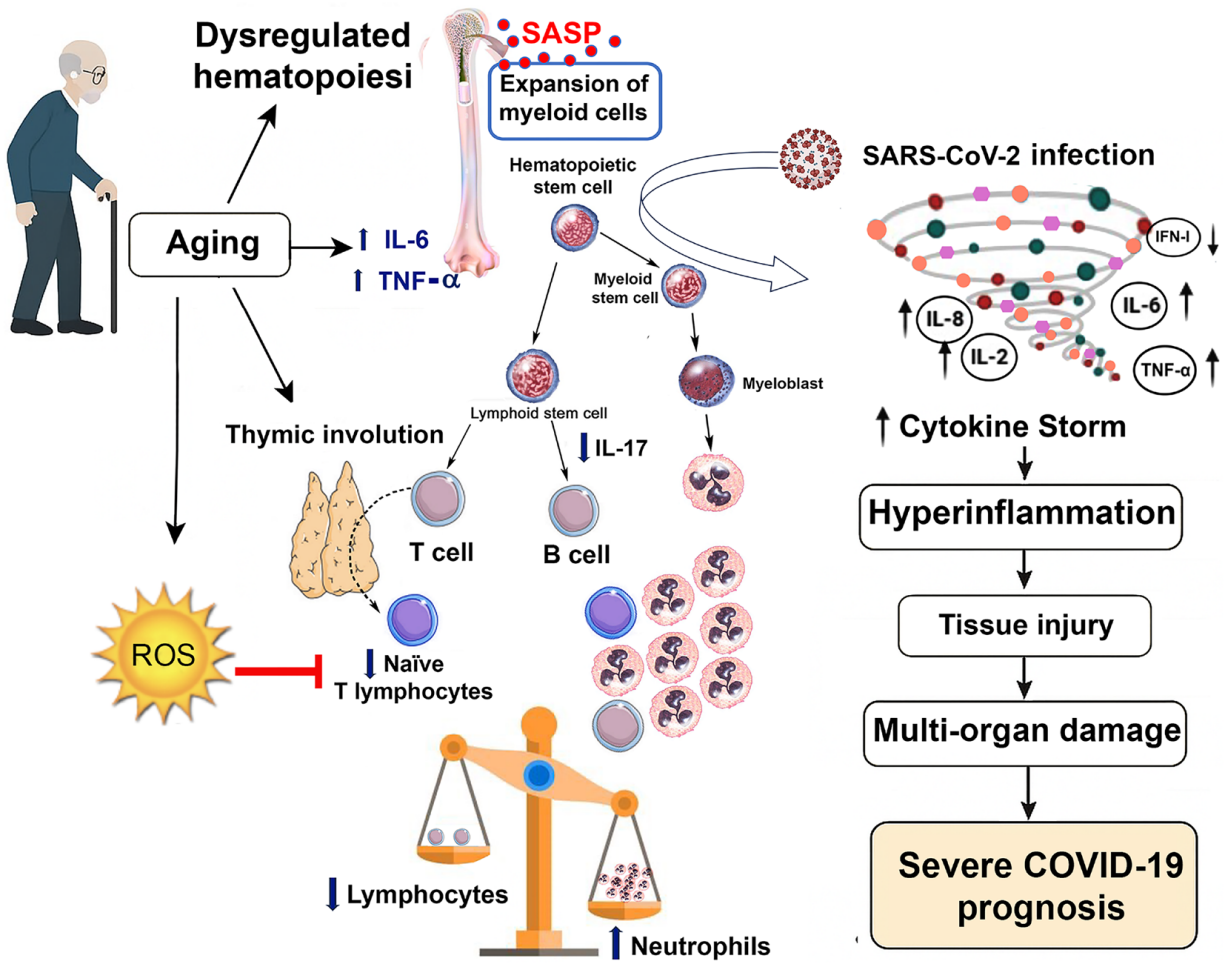
Schematic representation of the interplay between aging‐related lymphopenia, neutrophilia, and neutrophil‐to‐lymphocyte ratio (NLR) and COVID‐19 severity.

Evidence reviewed in this work highlights the consistent association of lymphopenia, neutrophilia, and elevated neutrophil‐to‐lymphocyte ratio (NLR) with poor outcomes in elderly COVID‐19 patients. Among these, NLR emerges as the most robust biomarker, as it integrates both adaptive decline and innate hyperactivation into a single measure (Fois et al., 2022; Henry et al., 2020; Yıldız et al., 2023). Elevated NLR mirrors systemic inflammation and immune dysregulation, and correlates with adverse outcomes, as depicted in Figure 5.

Lymphopenia, already a hallmark of immunosenescence, predicts frailty, prolonged hospitalization, and higher mortality across settings (Rubio‐Rivas et al., 2016; Zafrir et al., 2022; Zidar et al., 2019), while neutrophilia reflects the pro‐inflammatory state and dysregulated innate responses typical of aging (Hazeldine et al., 2014; Ince et al., 2024; Zhao et al., 2020).

Nevertheless, heterogeneity exists. Reported NLR cut‐off values vary widely, reflecting differences in populations, disease severity, and laboratory standards (Li, Liu, et al., 2020; Liu et al., 2020). Some cohorts confirm its independent predictive power (Di Rosa et al., 2023), while others note reduced accuracy when comorbidities or inflammatory conditions coexist (Bota et al., 2024). Evidence on sex differences in lymphocyte decline and inflammation is also emerging but remains inconclusive, with many studies underpowered to detect gender‐specific effects (Calabrò et al., 2023; Ghaab et al., 2024).

From a clinical perspective, these hematological parameters are inexpensive, rapid, and universally available. Their integration into geriatric practice could support early risk stratification, guide hospitalization and monitoring decisions, and complement frailty indices or comorbidity scores (Paganelli & Di Iorio, 2025; Pellegrino et al., 2024). In acute care, identifying elderly patients with marked lymphopenia or elevated NLR may facilitate timely escalation of care (Yoon & Lee, 2021; Sarengat et al., 2021), while in community‐dwelling older adults, persistently altered values may signal biological aging and immunological frailty (Navarro‐Martínez & Cauli, 2021; Zidar et al., 2019).

Lymphopenia, neutrophilia, and elevated NLR reflect biological aging and immune frailty (Navarro‐Martínez & Cauli, 2021; Paganelli & Di Iorio, 2025). These markers, linked to immunosenescence and chronic inflammation, indicate diminished immune response in the elderly (Liu et al., 2023; Santoro et al., 2021). The NLR quantifies this shift from adaptive to innate dominance (García‐Escobar et al., 2023). They serve as proxies for biological age, predicting frailty and infection risk (Crétel et al., 2011; Henry et al., 2020).

However, limitations must be acknowledged. Much of the available evidence is retrospective and hospital‐based, with heterogeneous designs and variable definitions of hematological thresholds (Ciccullo et al., 2020; Henry et al., 2020). Very old patients (>80 years) and frail subgroups are underrepresented, and longitudinal data on baseline hematological profiles prior to infection are scarce (Sturmlechner et al., 2025).

Future research should prioritize prospective studies in elderly cohorts, including the oldest‐old, women, and individuals with multimorbidity (Dennis et al., 1998; Niu et al., 2022). Standardization of NLR cut‐off values and integration with other markers of immunosenescence could refine predictive accuracy (Palmer et al., 2018; Valiathan et al., 2016). Interventional studies are also needed to assess whether monitoring or modulating these parameters can improve outcomes (Calabrò et al., 2023; Lewis et al., 2021).

## 5 CONCLUSION

Lymphopenia, neutrophilia, and elevated neutrophil‐to‐lymphocyte ratio (NLR) are reliable markers of poor prognosis in elderly COVID‐19 patients, reflecting immunosenescence and inflammaging. Their simplicity and predictive power make them valuable for early risk stratification in geriatric care.

This review integrates these biomarkers with the framework of aging‐related immune dysfunction, offering a new perspective on their role beyond prognostic value. However, comparisons between elderly and younger populations, as well as baseline aging markers, remain limited. Future studies should focus on prospective validation in frail populations, standardization of NLR cut‐offs, and exploring the role of these markers in broader aging contexts.

## FUNDING INFORMATION

This research received no external funding.

## ETHICS STATEMENT

This article is a literature review and did not involve the collection of primary data from human participants or animals. Therefore, ethical approval was not required.

## Supporting information


Data S1.
