# Supplementary material for: The neutrophil‐to‐lymphocyte ratio as a marker of immunosenescence and COVID‐19 outcomes in the elderly: A narrative review
Source: Physiol Rep. 2026 Feb 11;14(3):e70682. doi: 10.14814/phy2.70682 (PMC12894097; doi:10.14814/phy2.70682)
Supplement: Supplementary file 1 — Data S1. [file PHY2-14-e70682-s001.docx]

**2. Methods**

**2.1 Literature Search Strategy**

This narrative review was developed through a comprehensive literature search in PubMed/MEDLINE, Scopus, and Web of Science. Keywords and MeSH terms included: “COVID-19” OR “SARS-CoV-2”, AND “aging” OR “elderly” OR “older adults”, AND “immunosenescence”, AND “lymphopenia” OR “lymphocyte count”, AND “neutrophilia” OR “neutrophil count”, AND “neutrophil-to-lymphocyte ratio” OR “NLR”, AND “hematological biomarkers”. Boolean operators AND/OR were applied to combine concepts, ensuring comprehensive retrieval of relevant studies. Equivalent search strategies were adapted for Scopus and Web of Science, with filters for English language and human studies. This detailed approach allows reproducibility and ensures that key studies on age-related hematological changes in COVID-19 were systematically identified.

**2.2 Inclusion and Exclusion Criteria**

Inclusion criteria were human participants aged ≥60 years (or studies providing stratified data for elderly subgroups) with laboratory-confirmed SARS-CoV-2 infection, from community, hospital, or intensive-care settings. Studies included original research, systematic reviews, or meta-analyses reporting lymphopenia, neutrophilia, or NLR, as well as observational, clinical, or interventional designs. Mechanistic studies on immunosenescence, inflammaging, or immune dysregulation relevant to these markers were also considered. Only peer-reviewed articles in English were included.

Exclusion criteria were non-COVID-19 populations, animal/in vitro studies, narrative reviews without transparent methodology, duplicate datasets, non-English articles, studies without quantitative data on lymphocytes, neutrophils, or NLR, and studies focusing solely on other biomarkers (e.g., CRP, IL-6) without addressing the selected hematological parameters.

**2.3 Methodological Approach and Study Selection**

To minimize the risk of selection bias, the literature search and study selection were carried out independently by multiple researchers. This collaborative approach ensured an objective and transparent process. Studies were selected based on predefined inclusion and exclusion criteria, focusing on research related to lymphopenia, neutrophilia, and the neutrophil-to-lymphocyte ratio (NLR) in the context of aging and COVID-19. All studies were screened and assessed for eligibility by at least two researchers to ensure consistency and reduce subjectivity in the selection process.

While no studies were excluded based on contradictory findings, the search was exhaustive and aimed at identifying all relevant research. Despite a thorough review, no studies directly contradicted the relationship between lymphopenia, neutrophilia, and NLR with COVID-19 outcomes in older adults, suggesting a high degree of consistency in the literature rather than a bias in study selection.

To further enhance transparency and methodological rigor, we have included a **comparative table of threshold values for lymphocytes, neutrophils, and NLR** in different age groups. This table presents a summary of the threshold values found in the literature and reinforces the clinical relevance of these biomarkers in the context of COVID-19 outcomes in older adults. The inclusion of this comparative data adds depth to the analysis and strengthens the evidence base for the conclusions drawn.

Additionally, a PRISMA 2000 flow diagram (Figure 1) is provided to outline the number of studies identified, screened, and included in the final analysis, as well as the reasons for exclusion. This diagram ensures that the process of study selection can be easily followed and replicated.

Although a systematic review or meta-analysis could provide more robust evidence, the heterogeneous nature of the studies identified (in terms of population, study design, and methodology) made it difficult to perform such an analysis within the scope of this narrative review. However, we acknowledge that a future systematic review or meta-analysis would be valuable for consolidating and validating the findings from the studies identified, providing a more comprehensive synthesis of the existing evidence.

**Table S1. Comparative Data on Threshold Values for Lymphocytes, Neutrophils, and NLR**

| **Marker** | **Threshold for Younger Adults** | **Threshold for Older Adults** | **Clinical Implication** | **Reference** |
| --- | --- | --- | --- | --- |
| **Lymphocyte Count** | <1,000 cells/µL (moderate risk) | <1,000 cells/µL (strong predictor of severe outcomes) | Predicts severe COVID-19 outcomes in both groups, but more strongly in older adults | Liang et al., 2024 |
| **Neutrophil Count** | >7,500 cells/µL (associated with severe outcomes) | >7,500 cells/µL (associated with high mortality) | Increased neutrophil count is more significant in older adults | Zhou et al., 2020 |
| **NLR** | >3 (predictive of systemic inflammation) | >3 (strong predictor of poor prognosis) | NLR >3 is particularly predictive of worse outcomes in elderly patients | Paganelli & Di Iorio, 2025 |
